# Assessment of Point-of-Care Diagnostics for G6PD Deficiency in Malaria Endemic Rural Eastern Indonesia

**DOI:** 10.1371/journal.pntd.0004457

**Published:** 2016-02-19

**Authors:** Ari W. Satyagraha, Arkasha Sadhewa, Rosalie Elvira, Iqbal Elyazar, Denny Feriandika, Ungke Antonjaya, Damian Oyong, Decy Subekti, Ismail E. Rozi, Gonzalo J. Domingo, Alida R. Harahap, J. Kevin Baird

**Affiliations:** 1 Eijkman Institute for Molecular Biology, Jakarta, Indonesia; 2 Eijkman-Oxford Clinical Research Unit, Jakarta, Indonesia; 3 PATH, Seattle, Washington, United States of America; 4 Centre for Tropical Medicine, Nuffield Department of Medicine, University of Oxford, Oxford, United Kingdom; Johns Hopkins Bloomberg School of Public Health, UNITED STATES

## Abstract

**Background:**

Patients infected by *Plasmodium vivax* or *Plasmodium ovale* suffer repeated clinical attacks without primaquine therapy against latent stages in liver. Primaquine causes seriously threatening acute hemolytic anemia in patients having inherited glucose-6-phosphate dehydrogenase (G6PD) deficiency. Access to safe primaquine therapy hinges upon the ability to confirm G6PD normal status. CareStart G6PD, a qualitative G6PD rapid diagnostic test (G6PD RDT) intended for use at point-of-care in impoverished rural settings where most malaria patients live, was evaluated.

**Methodology/Principal Findings:**

This device and the standard qualitative fluorescent spot test (FST) were each compared against the quantitative spectrophotometric assay for G6PD activity as the diagnostic gold standard. The assessment occurred at meso-endemic Panenggo Ede in western Sumba Island in eastern Indonesia, where 610 residents provided venous blood. The G6PD RDT and FST qualitative assessments were performed in the field, whereas the quantitative assay was performed in a research laboratory at Jakarta. The median G6PD activity ≥5 U/gHb was 9.7 U/gHb and was considered 100% of normal activity. The prevalence of G6PD deficiency by quantitative assessment (<5 U/gHb) was 7.2%. Applying 30% of normal G6PD activity as the cut-off for qualitative testing, the sensitivity, specificity, positive predictive value, and negative predictive value for G6PD RDT versus FST among males were as follows: 100%, 98.7%, 89%, and 100% versus 91.7%, 92%, 55%, and 99%; P = 0.49, 0.001, 0.004, and 0.24, respectively. These values among females were: 83%, 92.7%, 17%, and 99.7% versus 100%, 92%, 18%, and 100%; P = 1.0, 0.89, 1.0 and 1.0, respectively.

**Conclusions/Significance:**

The overall performance of G6PD RDT, especially 100% negative predictive value, demonstrates suitable safety for G6PD screening prior to administering hemolytic drugs like primaquine and many others. Relatively poor diagnostic performance among females due to mosaic G6PD phenotype is an inherent limitation of any current practical screening methodology.

## Author Summary

G6PD is an enzyme that chemically protects us from otherwise toxic substances, like some chemotherapeutic agents. About 8% of people exposed to malaria have an inherited disorder that impairs G6PD activity, leaving them vulnerable to harm by an important therapy against malaria, primaquine. This drug alone prevents repeated clinical attacks stemming from dormant parasites residing in the human liver. Absent certain knowledge of patient G6PD status, healthcare providers managing patients infected by *Plasmodium vivax* or *Plasmodium ovale* malaria must choose between risk of harm caused by hemolytic toxicity of primaquine and that caused by the parasite after withholding therapy. Resolving that therapeutic dilemma requires assessment of patient G6PD status at the point-of-care in the impoverished rural tropics, where the vast majority of malaria patients live. Current technology for such screening is impractical in that setting. In this study we evaluated screening designed for practicality at the endemic tropical point-of-care: a rapid diagnostic test for G6PD (G6PD RDT; CareStart G6PD, AccessBio, USA). We found the G6PD RDT to be effective in screening volunteers living in rural eastern Indonesia. This G6PD RDT kit costs relatively little ($1.50), was simple to execute and interpret, required no specialized equipment or skills, performed well at ambient tropical temperatures (>30°C), and required no cold chain storage. This and similar kits may permit safe universal access to primaquine therapy against relapse of *P*. *vivax*, a vitally important step forward in mitigating the global burden of morbidity and mortality imposed by this pernicious parasite.

## Introduction

Glucose-6-phosphate dehydrogenase deficiency (G6PDd) is the most common inherited disorder, affecting about 400 million people [1–3]. G6PD enzyme catalyzes the first and rate-limiting reaction of the pentose phosphate pathway, the only means of reducing nicotinamide adenine dinucleotide phosphate (NADPH) in red blood cell cytosol. In turn, NADPH is the sole source of electrons for reducing glutathione, the principal means of maintaining healthy reduction-oxidation (redox) equilibrium in cytosol. Oxidative stress upon red blood cells with impaired G6PD activity leads to threatening redox imbalance. Most people with G6PDd nonetheless lead healthy lives of normal longevity, and it is only exposure to certain drugs, chemicals, foods or infections that impose hemolytic crisis and risk of serious harm. In the malaria endemic rural tropics, the most threatening scenario is becoming infected by the parasite *Plasmodium vivax* and being prescribed the drug primaquine to prevent the repeated clinical attacks (called relapses) deriving from latent liver stages called hypnozoites [2].

Primaquine is an 8-aminoquinoline drug licensed as a hypnozoitocide in 1952 and remains the only therapy available for preventing relapse [4]. During its clinical development in and after World War II, investigators observed hemolytic sensitivity in some subjects. Only later, in 1956, did those investigators identify deficiency in G6PD as the cause of that sensitivity [5]. Those early studies, conducted in prisoner volunteers in the USA, characterized primaquine sensitivity in African-Americans expressing the A- variant of G6PDd typically expressing 10–20% of normal G6PD activity. Primaquine hemolyzed only older red blood cell populations in those subjects and hemolysis ceased despite continued exposure to high doses of primaquine [6,7]. These findings led to the view of primaquine-induced hemolysis as relatively mild and self-limiting. However, studies in the 1960s revealed other variants of G6PDd, including the exquisitely primaquine-sensitive Mediterranean variant [8–12]. Mediterranean variant typically exhibited <5% of normal activity and primaquine-induced hemolysis occurred even among the youngest red blood cells—severe and unlimited hemolysis without cessation of dosing. Over the decades that followed, confirmed severe hemolytic crises and deaths due to primaquine toxicity in G6PD deficient patients accumulated [13–17]. In South and Southeast Asia, where more than 80% of vivax malaria attacks occur, the extraordinary diversity of G6PDd is dominated by Mediterranean-like, severely deficient variants [18,19].

Recent recognition of *Plasmodium vivax* as a pernicious infection and its multiple relapses a serious clinical and public health threat [20–22] elevated awareness of the problem of G6PDd as a very significant barrier to safe primaquine therapy [23,24]. Absent the ability to identify G6PDd patients among those infected by *P*. *vivax*, providers must choose between risk of harm caused by the drug and that caused by the repeated clinical attacks allowed by withholding the drug. Resolving this therapeutic dilemma requires identifying those at risk of harm with primaquine therapy and thus ensuring those not at risk obtain the enormous therapeutic benefit of primaquine.

The most widely used and recommended procedure for screening patients for G6PD deficiency, excluding newborn screening, is the fluorescent spot test (FST), described by G6PD pioneer Ernest Beutler in 1966 [25]. In a laboratory setting the test is relatively simple and inexpensive. However, in the setting of the impoverished rural tropics, the requirements for laboratory skills, refrigeration, specialized equipment, and high costs have excluded its availability to the vast majority of patients suffering malaria. Expert consensus defined practicality criteria for point-of-care G6PD diagnostics that included simplicity of use, ease of interpretation, no specialized equipment or cold chain, and relatively low cost [26–28]. Expert consensus also acknowledged that the availability of such robust devices where most malaria patients live is a key to the control and elimination of endemic *P*. *vivax* malaria [28].

In the current study, the performance of the CareStart G6PD (G6PD RDT, AccessBio, USA) device against the FST using quantitative spectrophotometric G6PD assay as diagnostic gold standard was compared among residents in a malaria-endemic area of rural eastern Indonesia. The G6PD RDT performed as well as the FST.

## Methods

### Ethics Statement

This study has been ethically approved by the Eijkman Institute Research Ethics Commission (EIREC) (project No. 69, February 13^th^, 2014). After obtaining the informed consent from 610 healthy subjects at least 6 years old, recruitment ceased at targeted full enrollment. Written informed consent was obtained from all study participants. Parents or guardians signed the informed consents for minors under 18 years of age.

### Population and Study Site

**Fig 1:**
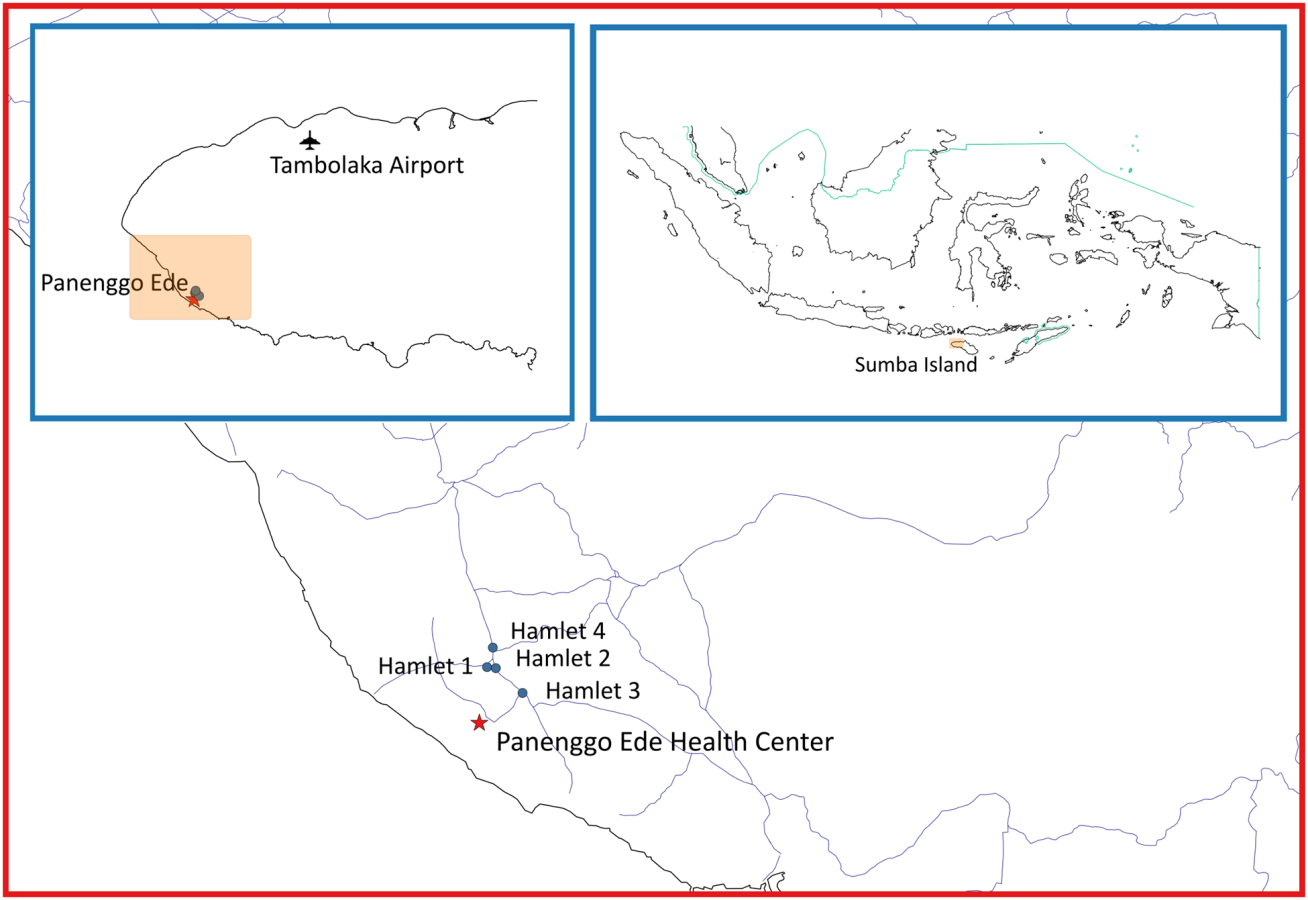
Geographic location of the study site.

**Fig 2:**
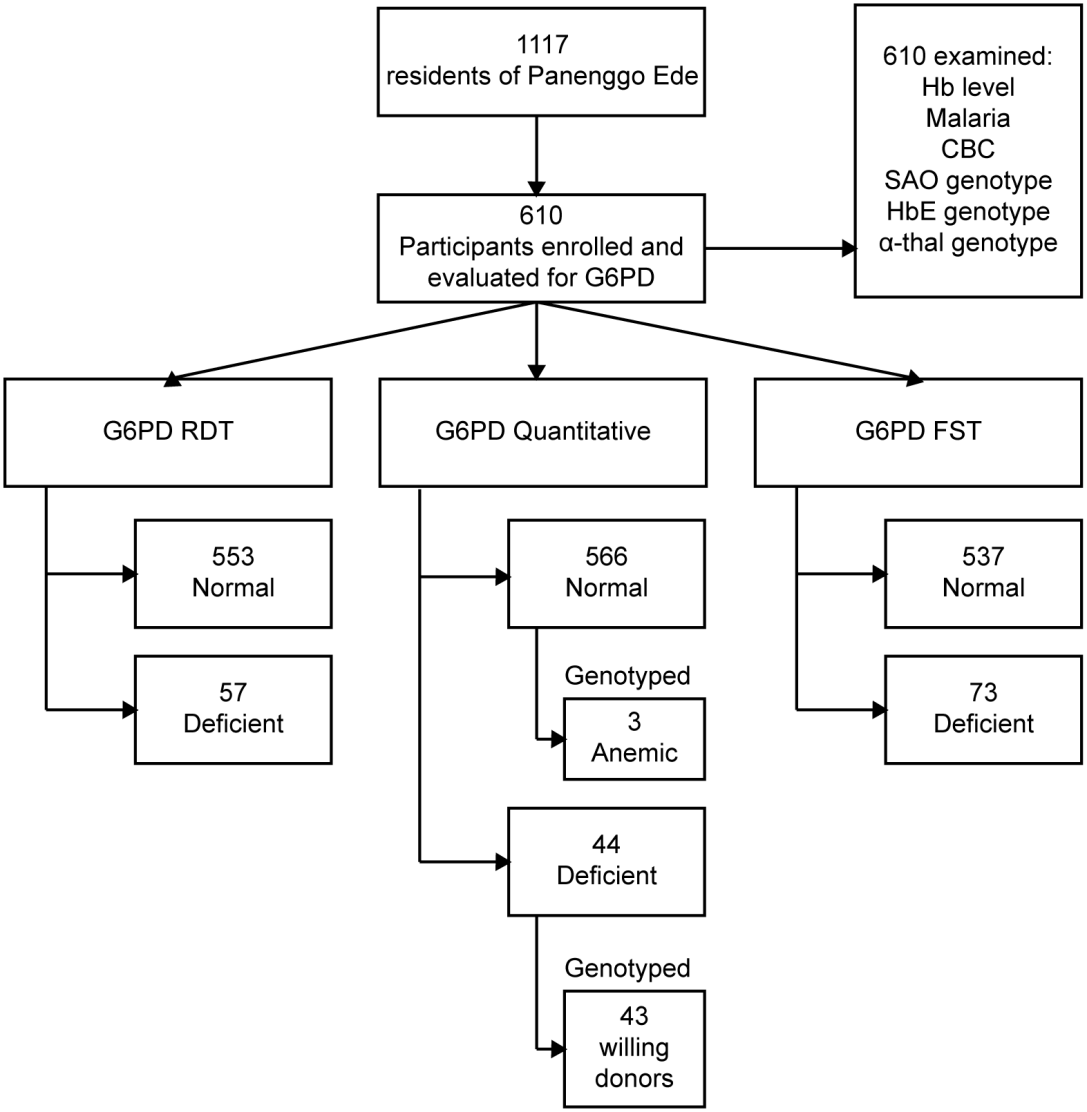
Flow-chart of the study in Panenggo Ede where those tests performed in field or field laboratory were confirmed by DNA analysis at the Eijkman Institute in Jakarta, Indonesia.

The village Panenggo Ede is located in the western coastal region of Southwest Sumba regency (Fig 1), where G6PDd prevalence was known to be >5% [29]. A total of 1117 people resided in this village. Fig 2 shows the work flow where the research team engaged the community gathered at churches or other social functions, and explained the study procedures and intent. Residents were then invited to a study center established in the village at designated times and dates between April and May 2014. The inclusion criteria were people ≥6 years old, healthy and willing to sign informed consent. A total of 350 females and 260 males provided a 3mL sample of whole venous blood collected into tubes containing EDTA anticoagulant. Samples were held at 4°C prior to processing and analysis on-site (G6PD RDT) or nearby temporary laboratory (FST) within 3 hours on the same day, or within 3 days at the laboratory in Jakarta (quantitative G6PD).

### G6PD RDT

**Fig 3:**
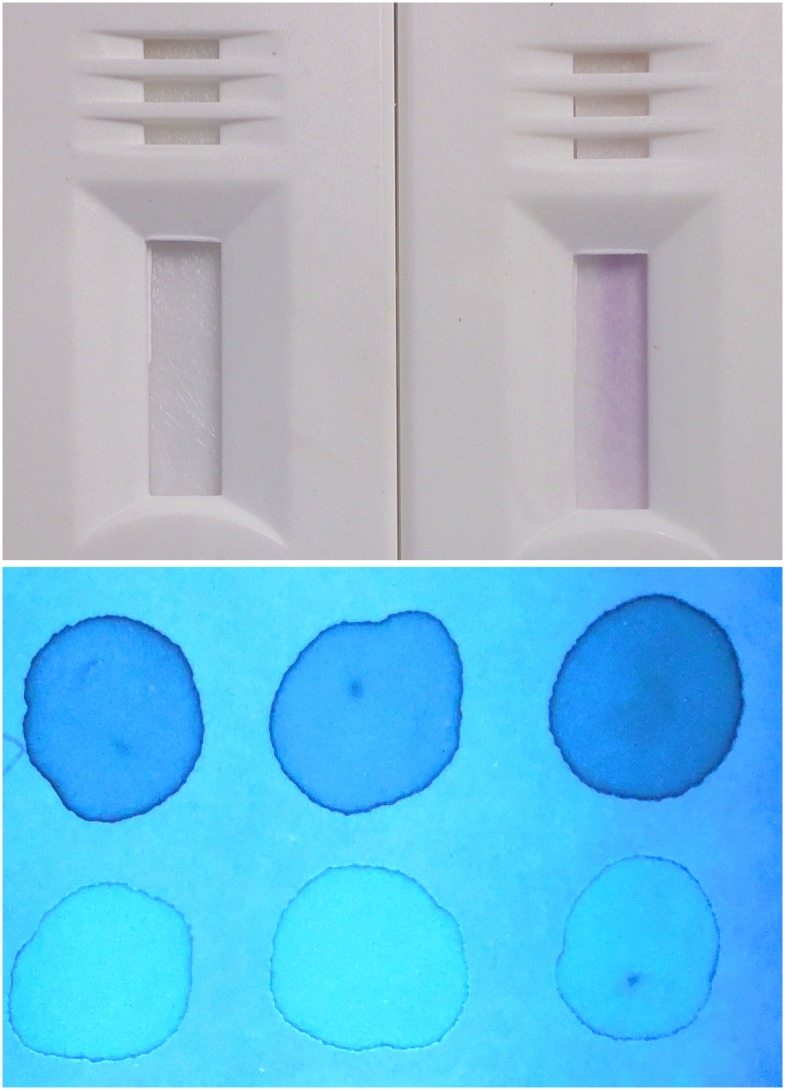
Photographs illustrating visual test outcomes for the G6PD RDT (top) and FST (bottom). For FST, samples were spotted at time 0, 5 and 10 minutes interval and the dark spots were considered deficient (D) and the bright ones were considered normal (N). RDT with purple color was considered normal (N) and no color was considered deficient (D).

The principle of the CareStart G6PD ^T^ screening test is reduction of a colorless nitro-blue tetrazolium dye to purple colored formazan. Thus, whereas a colorless test outcome indicates G6PD deficiency, a purple color reflects G6PD activity (Fig 3). Readers of the test were instructed to consider only a diagnosis of deficient or normal, with the demand to classify as deficient any test strip exhibiting a colorless to distinctly lighter hue of purple compared to that of most other tests. This approach would ensure safety when primaquine therapy would follow the diagnosis of G6PD normal.

Two microliters of whole blood was removed from the EDTA tube by a stick device included in the RDT kit and placed into the sample window, immediately followed by two drops of a provided buffer solution into the assay window according to the manufacturer’s instructions. After ten minutes at the ambient temperature of approximately 30°C, the RDT was visually read and classified as deficient or normal.

### Fluorescent Spot Test

At the end of each day of work in the village, venous blood was transferred on ice packs to a field laboratory in Weetabula to conduct the fluorescent spot test (FST, Trinity Biotech, Ireland; Cat. No. 203-A) using deficient (Cat. No. G5888), intermediate (Cat. No. G5029) and normal (Cat. No. G6888) G6PD controls from the same company. This qualitative test is a modification of Beutler’s test in which glucose-6-phosphate and NADP^+^ reagents (substrate solution) in the presence of G6PD sample produce fluorescent NADPH and 6-phosphogluconate. Progress of the reaction was observed in the dark under long-wave ultraviolet illumination of sample filter paper (Whatman No. 1 filter paper, Cat. No. 1001–150) at intervals of zero, 5 and 10 minutes. Briefly, 200 μl of substrate solution and 10 μl of gently mixed venous blood was put into a 5 ml tube and mixed by manual swirling. A single drop of this solution was transferred onto filter paper marked as time zero. The tube was then placed into a 37°C water bath for 5 min, when another drop was placed onto filter paper marked as time 5 min. This was repeated for the final sample at 10 min. The filter papers were allowed to dry at room temperature (25°-29°C) before visual inspection under UV light in an otherwise dark room. Deficient (no fluorescence), intermediate (weak fluorescence) and normal (strong fluorescence) controls were done for every set of 10 samples from the subjects. Readers were instructed to classify intermediate test outcomes as deficient.

### G6PD Quantitative Test

The principle of the G6PD quantitative assay from Trinity Biotech (Cat. No. 345-B) is similar to the FST. Fluorescence from NADPH produced in the same substrate solution mixture was read at 340nm using a high-grade, temperature-controlled spectrophotometer (UV-1800 UV-VIS Shimadzu). The same G6PD controls from Trinity Biotech were conducted for each set of 25 samples from the subjects. The assay was performed in an air-conditioned (~25°C) laboratory at the Eijkman Institute in Jakarta within 3 days of blood withdrawal. The venous blood tubes were kept at 4°C at all times prior to use in Jakarta. Hemoglobin level was determined using 10 μl of blood into a micro-cuvette supplied by the manufacturer of the HemoCue system (HemoCue AB, Sweden) and immediately read in the instrument (Hb201+) of that system for hemoglobin measurement prior to the G6PD quantitative assay. The manufacturer’s instructions were strictly followed for measuring absorbance at 340nm and deriving an estimate of G6PD activity in U/g Hb at 30°C using the incubated spectrophotometer. Although the manufacturer recommended a cut off value <4.6 U/g Hb for deficient activity, we selected <5U/g Hb on the basis of prior survey in the same area showing a median G6PD activity of 10 U/g Hb [29]. We aimed for a 50% cut off value, that being the limit of relative safety with respect to potential hemolytic loss of red blood cell populations, knowing this value correlated with the proportion of deficient red blood cells in a laboratory model of the female heterozygous state [30]. The assay was performed in triplicates where a mean was derived to be used for downstream analyses.

### G6PD Variant Genotyping

Samples for G6PD genotyping were selected on the basis of a deficient classification by G6PD quantitative assay (<5U/gHb), or by having Hb <8g/dL (Fig 2). DNA from the buffy coat of venous blood in EDTA tubes was extracted using QIAamp DNA Blood Mini Kit (Qiagen, Cat. No. 51106). DNA from subjects classified as deficient by quantitative G6PD assay was examined by PCR/RFLP for the most common variants in Sumba: Vanua Lava, Viangchan, Chatham, and Kaiping. Table 1 details the primers employed and the PCR and RFLP products thus expected. PCR conditions were as follow: 1X buffer GC Hifi (Kapa Biosystem), 200 μM dNTPs, 200 μM forward and reverse primer each, 0.4 U Kapa Hifi polymerase in 25 μl PCR reaction. PCR cycle for the variants were also the same except in the annealing temperature: 95°C 5 min before entering PCR cycle of 30X; denaturation at 95°C for 30 sec; and annealing 65°C, 56°C, 61°C and 62°C, for Vanua Lava, Viangchan, Chatham and Kaiping respectively. Each was followed by extension at 72°C for 30 sec, where another 7 min at 72°C was needed at the end of the 30 cycles. The PCR products were cut with the restriction enzymes as listed in Table 1. After incubation at 37°C overnight, products were run on 3% agarose gel for analysis. The samples testing as normal for the common variants were PCR and whole-gene sequenced as described by others [31]. Sequences were aligned to G6PD reference sequence from NCBI, NG_009015.2.

**Table 1:** PCR primers and RFLP conditions for G6PD variants common in Southwest Sumba Regency and PCR primers for detecting SAO, HbE and α thalassemia.

Variant	Primer	Primer Sequence (5’ → 3’)	Expected PCR Product (bp)	RE	Expected Result (bp)		References
					Deficient	Normal	
**Vanua Lava**	VL-9F	CAG CCT GGG GCA GTG TCT GTG CT	366	*Eco*NI	366	346	Our design
	VL-9R	GCG GTT GGC CTG TGA CCC CTG GTG				20	
**Viangchan**	VC-9F	TGG CTT TCT CTC AGG TCT AG	126	*Xba*I	106	126	Nuchprayoon *et al*, 2002
	VC-9R	GTC GTC CAG GTA CCC TTT GGG G			20		
**Chatham**	CT-9F	CAA GGA GCC CAT TCT CTC CCT T	208	*Bst*XI	100	130	Gandomani *et al*, 2011
	CT-9R	TTC TCC ACA TAG AGG AGG ACG GCT GCC AAA GT			78	78	
					30		
**Kaiping**	KP-9F	ACG TGA AGC TCC CTG ACG C	227	*Mnl*I	206	227	Laosombat *et al*, 2006
	KP-9R	GTG CAG CAG TGG GGT GAA CAT A			21		
SAO	OVF 1098	GGG CCC AGA TGA CCC TCT TGC	175 (148 for SAO)	-	-	-	Jarolim *et al*, 1991
	OVR 1272	GCC GAA GGT GAT GGC GGG TG					
HbE	Com C	ACC TCA CCC TGT GGA GCC AC	293	*Mnl*I	122	106	Pramoodjago *et al*, 1999
	TLR 62320	CTA TTG GTC TCC TTA AAC CTG TCT TGT AAC CTT GCT A			106	60	
alpha Thalassemia (Multiplex PCR—2 gene deletion)	SEA-alpha F	CTC TGT GTT CTC AGT ATT GGA GGG AAG GAG	1110 660	*-*	-	-	Liu *et al*, 2000
	SEA-alpha R	ATA TAT GGG TCT GGA AGT GTA ACC CTC CCA					
	alpha R	TGA AGA GCC TGC AGG ACC AGG TCA GTG ACC G					
	FILL-alpha F	AAG AGA ATA AAC CAC CCA ATT TTT AAA TGG GCA	550	*-*	-	-	
	FILL-alpha R	GAG ATA ATA ACC TTT ATC TGC CAC ATG TAG CAA					
	THAI-alpha F	CAC GAG TAA AAC ATC AAG TAC ACT CCA GCC	411	*-*	-	-	
	THAI-alpha R	TGG ATC TGC ACC TCT GGG TAG GTT CTG TAC C					
α Thalassemia (Multiplex PCR—1 gene deletion)	2/3P	TGT TGG CAC ATT CCG GGA CAG	1940 (normal)	*-*	-	-	Setianingsih *et al*, 2003
	XY1	GCG CCG AGC CTG GCC AAA CCA TCA CTT TTC	2220 (-3.7 kb deletion)				
	3R1	TGC ATC CTC AAA GCA CTC TAG GGT CCA GCG T	1673 (-4.2 kb deletion)				
	SA3P	TAA GCT AGA GCA TTG GTG GTC ATG C					
	XYHA	GAA GTA CGT CCG ACC AGC TTA GCC A					

RE is restriction enzyme; bp is base pair.

### Red Cell Disorder Genotyping

DNA extracted from venous blood was also genotyped for Southeast Asian ovalocytosis (SAO), alpha thalassemia, and hemoglobin E (HbE). Table 1 lists the primers for SAO, one and two gene deletions for alpha thalassemia and HbE. The PCR conditions for each mutation were as reported elsewhere [32]. PCR conditions for one-gene deletions were as previously reported [33].

### Analytical Rationale and Statistics

In the current study the diagnostic objective was not G6PD deficiency *per se*, but a diagnostic outcome indicating either hazard or safety with administration of a potentially hemolytic drug. As such, diagnostic performance of the G6PD screening techniques was linked to the perceived primaquine safety margin of 30% of normal activity per WHO recommendation [26]. We aimed to classify all male hemizygotes and female heterozygotes having less than variable thresholds of normal G6PD activity (<10%, <30%, or <60%) as deficient. The median G6PD activity among subjects having ≥5U/g Hb was considered 100% of normal. These thresholds represented an examination of variance in diagnostic performance representing poor, good, and complete safety, respectively, with respect to exposure to primaquine. Poor safety at 10% would likely include patients at risk of hemolysis, whereas complete safety at a 60% would unnecessarily deny some patients primaquine treatment. The 30% cut off value represents a compromising balance of those problems.

Diagnostic performance of the qualitative G6PD RDT and FST were assessed against the quantitative G6PD classification as “deficient” or “normal” at G6PD activity thresholds. Further, the analyses were segregated by sex for the simple reason that hemizygosity versus heterozygosity (males and females, respectively) profoundly impacts diagnostic performance for G6PD deficiency [34]. Males tend to be wholly deficient or normal, whereas females will present the full spectrum of G6PD activity levels due to mosaicism of this X-linked trait [35].

Standard methods for calculation of sensitivity, specificity, positive predictive value, and negative predictive value were applied to the G6PD RDT and FST for each threshold of percent of normal G6PD activity. The meaning of these parameters in the context of a diagnosis guiding primaquine therapy bears explanation here. Sensitivity and specificity are easily grasped, i.e., rate of true positives and rate of true negatives, respectively. The terms “positive” and “negative” refer to what is defined here as “deficient” and “normal” G6PD phenotype, respectively. A test negative for G6PD activity is positive for G6PD deficiency, and vice versa for a positive test for G6PD activity. The terminology “deficient” (positive for deficiency) versus “normal” (negative for deficiency) recommended by WHO [26], avoids confusion and was adopted here. Further, the terms “deficient predictive value” (DPV) and “normal predictive value” (NPV) were applied for consistency and clarity, but using precisely the same standard mathematical methods for all of these statistics.

**Fig 4:**
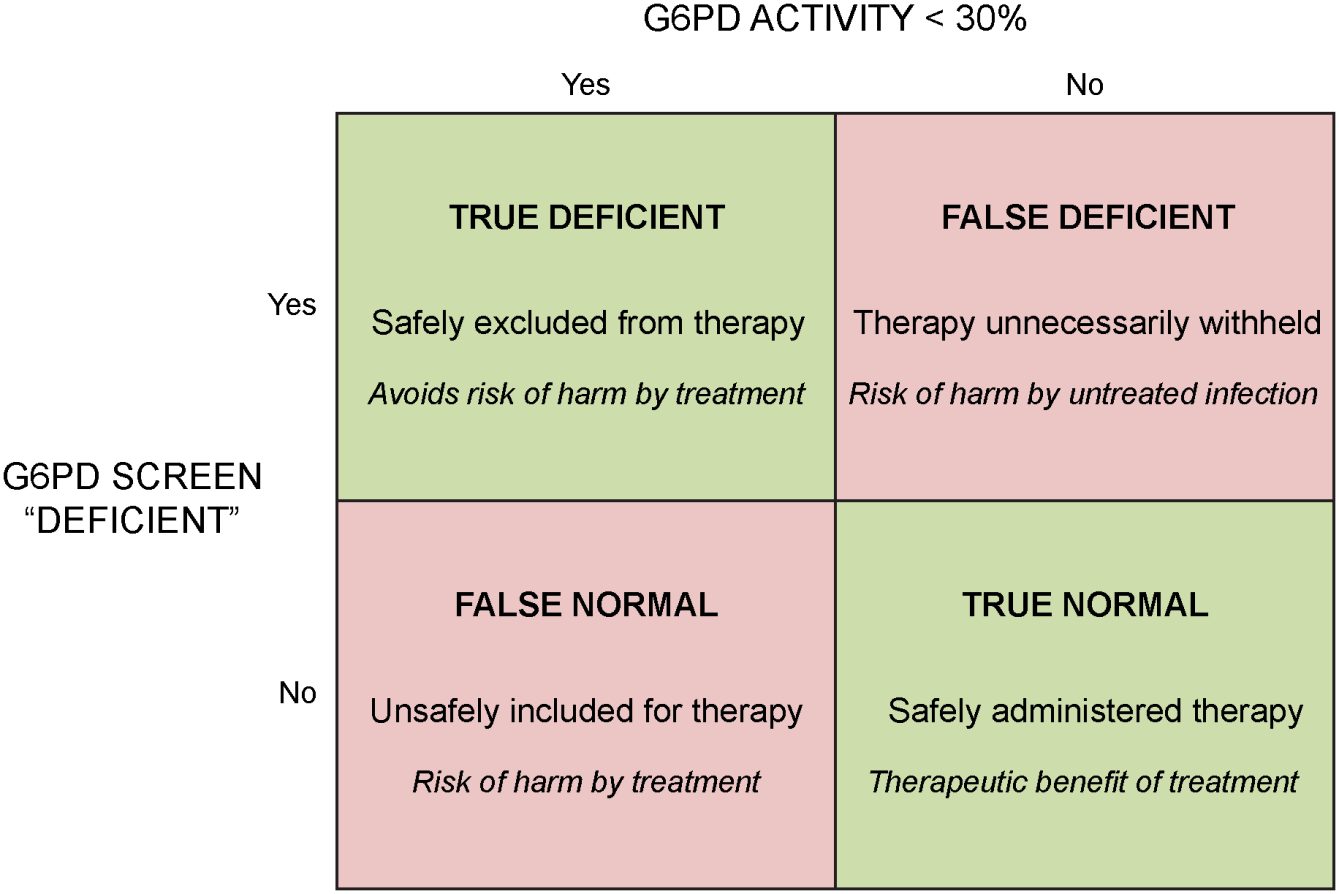
Chart illustrating the rationale for assessing diagnostic performance of qualitative G6PD screening devices in the context of a clinical decision to offer or withhold primaquine therapy in patients with *P*. *vivax* malaria. Each classification (bold font top), clinical outcome (normal font middle), and risk or benefit (italics bottom) of diagnostic performance appears in each box of classification.

DPV estimates the probability that those classified as deficient truly are, whereas NPV estimates the probability that those classified as normal truly are. In the primaquine therapy context of G6PD diagnostics, the most important statistic is NPV because a diagnosis of normal prompts exposure to primaquine. Fig 4 illustrates the rationale at work. In a practical sense, NPV estimates the probability of primaquine being safely administered, whereas DPV reflects the proportion of patients being denied primaquine therapy who actually cannot take it safely.

Statistical significance of diagnostic performance indicator by diagnostic test was evaluated by Chi-square test. Sensitivity, specificity, DPV and NPV were presented using proportion analysis and Fisher’s exact 95% confidence intervals. Mean and range of hemoglobin level were calculated to determine distribution by gender. Data were analyzed using Stata 9.

## Results

### Inherited Blood Disorders in the Community

The overall prevalence of G6PDd at Panenggo Ede by quantitative assay (<5U/g Hb) was 7.2% (44/610), 9.2% for males (24/260) and 5.7% (20/350) for females. Southeast Asian ovalocytosis (SAO) occurred in 12.7% (78/610), alpha thalassemia (alpha Thal) in 15.1% (92/610), and hemoglobin E (HbE) in 16.4% (100/610) of residents. Double mutations occurred among 13 residents having G6PDd (5 with SAO, 3 with alphaThal, 5 with HbE), and one subject had G6PDd, SAO and HbE. SAO occurred in 21 subjects also having alpha Thal, and in 7 people also having HbE. In total, 44.3% (270/610) of the population had one or more of these four blood disorders.

### Malaria and Anemia

Table 2 summarizes findings of malaria and anemia in the community. The overall prevalence of microscopically patent parasitemia was 2.5% (15/610); with 53% *P*. *falciparum*, 33% *P*. *vivax*, and 14% mixed by these species. The mean level (and range) of Hb in the study population was 13.2(6.0–22.8) g/dL. Only 3 subjects had levels <8.0g/dL, and the majority had ≥10.0g/dL (607; 99.5%). Males and females had similar but statistically distinct levels of Hb: 13.8 (6.9–20.2), and 12.8 (6.0–22.8), respectively (P<0.0001). Among the three severely anemic subjects (<8.0g/dL), genotyping for G6PD variants revealed one as a female (Hb 6.0g/dL) heterozygous for Vanua Lava variant with a quantitative G6PD value of 13.55 U/gHb. The other two were G6PD normal genotype and phenotype. Hemoglobin level did not appear to be significantly different between subjects with or without any particular inherited blood disorder evaluated.

**Table 2:** Malaria and anemia in the community.

Criteria	Total Subject	Female	Male
**Malaria**	15	7	8
*P*. *falciparum*	8	3	5
*P*. *vivax*	5	2	3
Mix (Pf + Pv)	2	2	0
**Hb**	610	350	260
Mean (range)	13.2 (6.0–22.8)	12.8 (6.0–22.8)	13.8 (6.9–20.2)
< 8 g/dL	3	2	1
Mean (range)	6.9 (6.0–7.8)	6.9 (6.0–7.8)	6.9
>10 g/dL	607	348	259
Mean (range)	13.4 (10.3–22.8)	13.0 (10.3–22.8)	13.9 (10.3–20.2)

### G6PDd Characteristics

**Fig 5:**
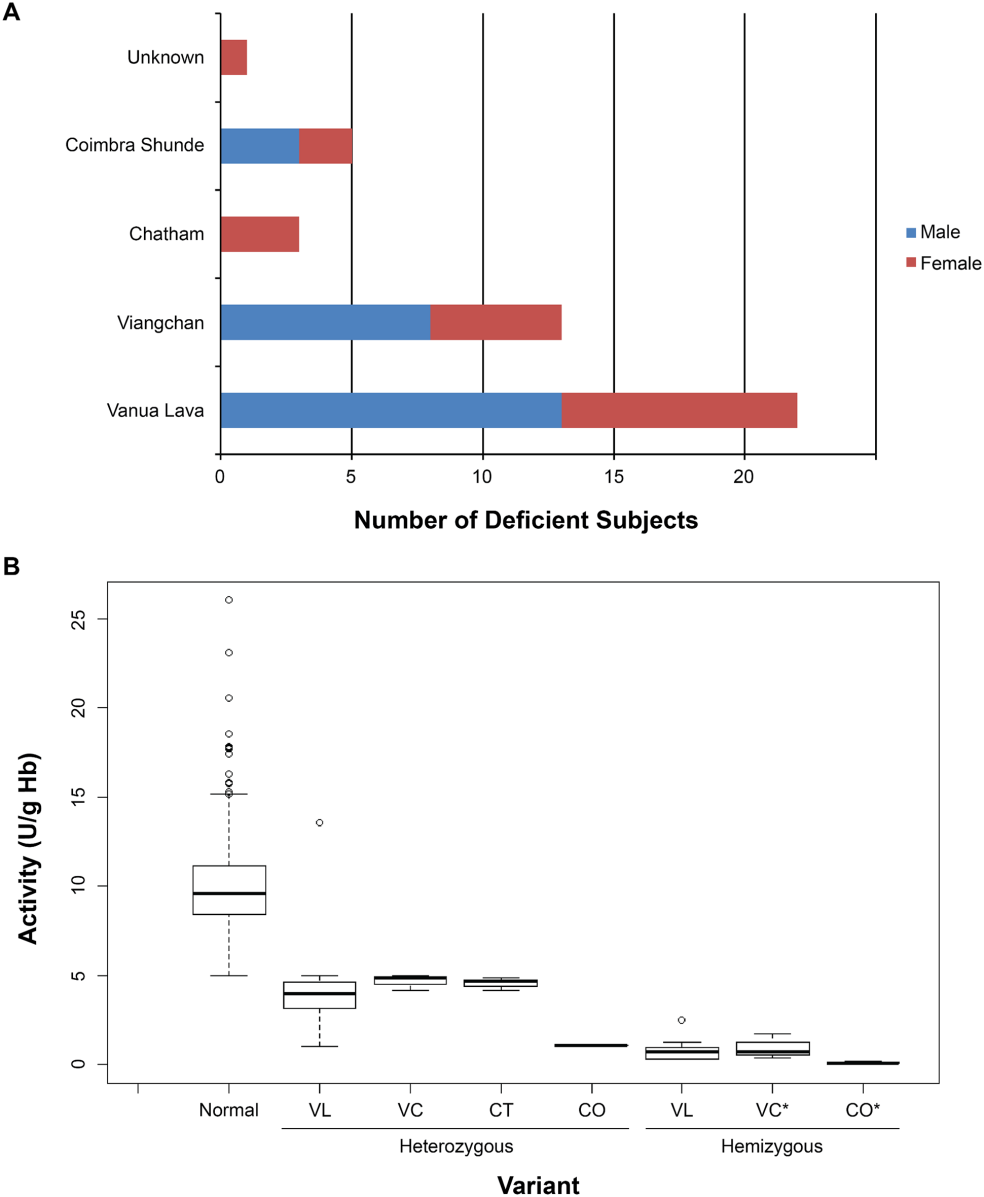
Variants of G6PD found in Panenggo Ede. (A) Bar graph showing different variants found in males (blue) and females (red) and (B) showed the boxplot showing the activity of these variants in comparison to normal. VL, VC, CT and CO stands for Vanua Lava, Viangchan, Chatham and Coimbra Shunde variant of G6PD respectively. Black line across each box plot is the median for each group. * Indicated that the group contained homozygous females as well.

Fig 5A illustrates the results of genotyping of the 44 subjects deemed G6PDd by quantitative assay (<5.0 U/g Hb). Vanua Lava dominated at 50% (22/44), followed by Viangchan at 30% (13/44), Coimbra Shunde at 11% (5/44), Chatham at 7% (3/44), and 1 subject was not successfully genotyped (2%). Fig 5B illustrates G6PD activity values for subjects classified as normal by G6PD activity, as well as with those classified as deficient and successfully genotyped. Heterozygous females having ≥5.0 U/gHb would have been excluded from the genotyping survey and would be included among normals in the figure. The values illustrated for heterozygotes inform only the diagnostic assessment rather than as a survey of their G6PD activity ranges. Among hemizygous males, however, the G6PD activity mean and range may be considered estimates of residual enzyme activity among the specific variants: 0.8 U/g Hb (0.27–2.5 U/g Hb) for Vanua Lava; 0.97 U/g Hb (0.52–1.62 U/g Hb) for Viangchan; and 0.09 U/g Hb (0.03–0.16 U/g Hb) for Coimbra Shunde. Remarkably low G6PD activity was also observed in the two females expressing Coimbra Shunde variant (0.57 U/g Hb; the mean of 0.11 and 1.04 U/g Hb). Chatham variant was found only in 3 females. G6PD activity did not vary with age in this study, as reported in another study from the same region [29].

### Diagnostic Assessment

**Fig 6:**
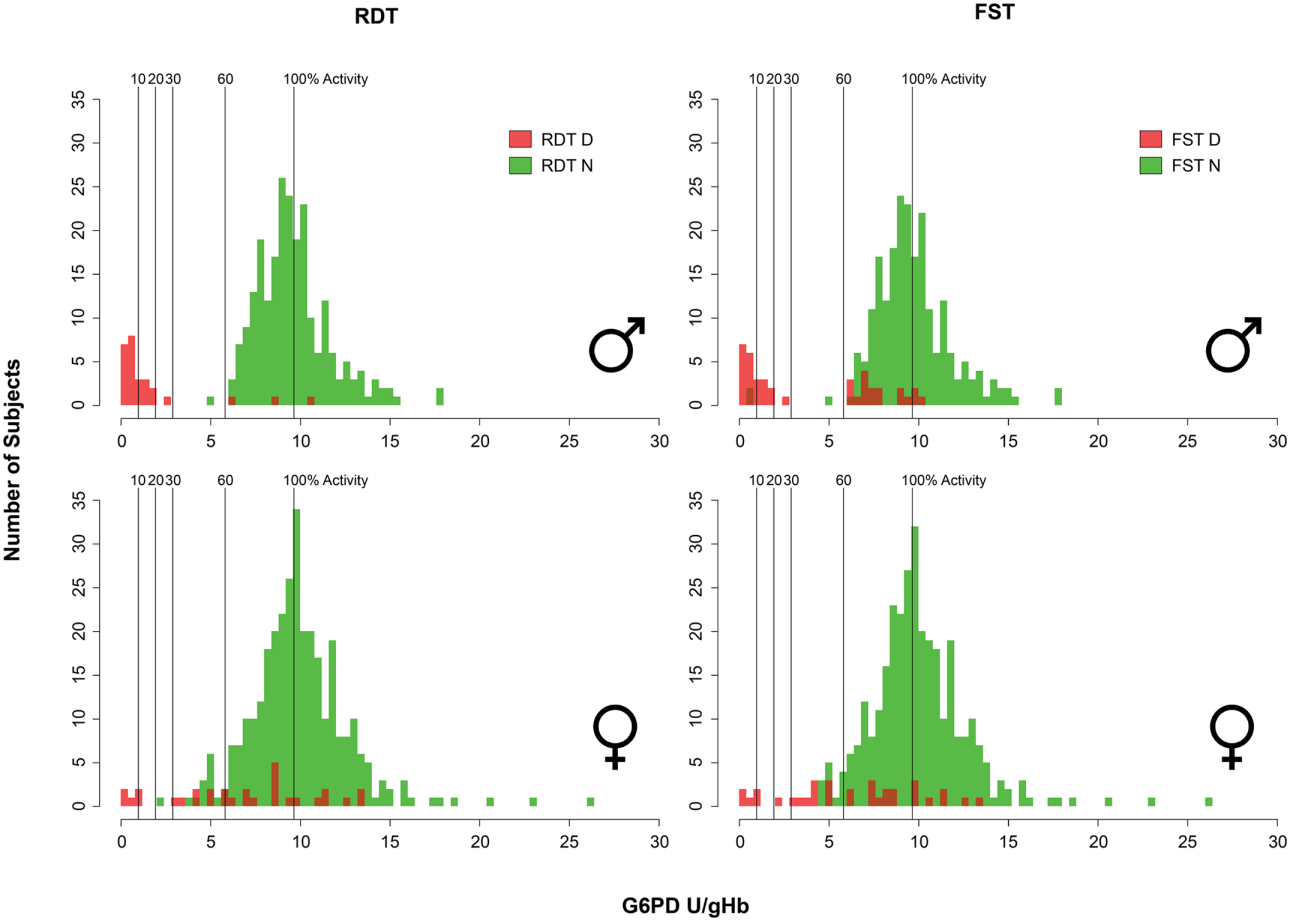
Graphs illustrate diagnostic outcomes for the G6PD RDT (left) and FST (right) among males (top) and females (bottom) across quantitative G6PD activity values for each subject. A qualitative test classification as deficient is shown in red, and green for normal. Vertical lines within each identify specific diagnostic thresholds (10%, 20%, 30%, 60% and 100%) employed to calculate diagnostic performance characteristics.

Fig 6 illustrates diagnostic outcomes for the G6PD RDT and FST across quantitative G6PD activity values in males and females. The tests performed similarly, with each discerning G6PD deficiency at a threshold of 10% of normal activity among males and females. However, 2 FST tests in males were read as normal at <10% of activity. The tests also performed similarly at a 60% activity threshold for both tests. The FST in males showed a propensity for false deficient reads, even at or above 100% of normal activity, but was especially frequent between 65% and 85% of normal activity. Three false deficient reads occurred among males with the G6PD RDT at 65%, 90%, and 115% of normal G6PD activity. Although both tests properly identified all female heterozygotes below a 30% threshold (with a single exception for the G6PD RDT at 22% of normal activity), each also exhibited a profound propensity for false deficient reads all across the range of G6PD activity values.

Table 3 summarizes the statistical analyses of these diagnostic outcomes among males and females diagnostic thresholds of 10%, 30%, and 60% for both of the qualitative G6PD screening kits. At the 30% threshold the G6PD RDT showed superior sensitivity and specificity in males compared to the same for the FST: 100% and 98.7% versus 91.7% and 92.4%, respectively (P = 0.48 and P < 0.001 for sensitivity and specificity respectively). Among females at the 30% threshold, no statistically significant differences occurred between the sensitivities and specificities of the two kits: 83.3% and 100% vs. 92.7% and 92.2% (P = 1 and P = 0.89) for G6PD RDT vs. FST, respectively. Deficient predictive value (DPV) for the G6PD RDT for males at 30% of normal G6PD activity was superior to the same with FST: 63.0% versus 37.5% (P = 0.05), respectively. Among females at the same threshold, DPV was 10.0% and 9.1% (P = 1). Among males for both G6PD RDT and FST at 30% threshold, normal predictive value (NPV) was 100% and 99.1% respectively (P = 0.23), and for females 100% and 100% (P = 1).

**Table 3:** G6PD diagnostic tests analyzed in 610 subjects living in Panenggo Ede village, Southwest Sumba, tested against G6PD gold standard test.

Cut off	Performance indicator ^1^	Male		P-value	Female		P-value
		RDT ^2^	FST ^3^		RDT	FST	
10%	Sensitivity	100.0 (80.5–100)	88.2 (63.6–98.5)	0.48	100.0 (29.2–100)	100.0 (29.2–100)	1.0
	Specificity	95.9 (92.6–98.0)	89.7 (85.2–93.2)	**0.01**	92.2 (88.9–94.8)	91.4 (87.9–94.1)	0.78
	DPV	63.0 (42.4–80.6)	37.5 (22.7–54.2)	0.05	10.0 (2.1–26.5)	9.1 (1.9–24.3)	1.0
	NPV	100.0 (98.4–100)	99.1 (96.8–99.9)	0.23	100.0 (98.9–100)	100.0 (98.8–100)	1.0
30%	Sensitivity	100.0 (85.8–100)	91.7 (73–99)	0.49	83.3 (35.9–99.6)	100.0 (54.1–100)	1.0
	Specificity	98.7 (96.3–99.7)	92.4 (88.2–95.4)	**0.001**	92.7 (89.5–95.2)	92.2 (88.8–94.8)	0.89
	DPV	88.9 (70.8–97.6)	55.0 (38.5–70.7)	**0.004**	16.7 (5.6–34.7)	18.2 (7–35.5)	1.0
	NPV	100.0 (98.4–100)	99.1 (96.8–99.9)	0.24	99.7 (98.3–100)	100.0 (98.8–100)	1.0
60%	Sensitivity	96.0 (79.6–99.9)	88.0 (68.8–97.5)	0.61	44.0 (24.4–65.1)	60.0 (38.7–78.9)	0.39
	Specificity	98.7 (96.3–99.7)	92.3 (88.2–95.4)	**0.001**	94.2 (91–96.4)	94.5 (91.4–96.7)	1.0
	DPV	88.9 (70.8–97.6)	55.0 (38.5–70.7)	**0.004**	36.7 (19.9–56.1)	45.5 (28.1–63.6)	0.61
	NPV	99.6 (97.6–100)	98.6 (96.1–99.7)	0.36	95.6 (92.8–97.6)	96.8 (94.3–98.5)	0.53

^1^ Proportion and 95% confidence interval.

^2^ CareStart RDT (Access Bio) and tested in field setting with temperature 28–34°C and humidity between 55–76%.

^3^ FST (Trinity Biotech) and tested in field laboratory with temperature 26–29°C.

## Discussion

This assessment of a new RDT for G6PDd (CareStart G6PD) revealed performance characteristics essentially similar to the current screening standard, the FST. Whereas the G6PD RDT meets essential performance characteristics defined by expert consensus [26–28], the FST meets almost none of those. The availability of practical G6PD diagnostic devices at the periphery of healthcare delivery in the endemic tropics would meet an urgent need to provide primaquine therapy to the G6PD-normal majority infected by the relapsing malarias [23, 24, 28, 36]. Consistency in satisfactory diagnostic performance of the G6PD RDT should impel making it broadly available in order to resolve the therapeutic dilemma of primaquine, G6PD deficiency and *P*. *vivax* or *P*. *ovale* malarias.

A study of a Cambodian population (n = 938) having 7.9% G6PD deficiency dominated by the Viangchan variant (92%) reported good performance of the G6PD RDT relative to the FST [37]. Investigators in Ghana also reported satisfactory performance of the G6PD RDT in a population (n = 206) dominated by the A- variant [38], as well as a study (n = 456) in Haiti [39]. All of these studies applied a quantitative diagnostic threshold of <30% of normal G6PD. Concordance among these studies offers assurance of satisfactory diagnostic performance of the G6PD RDT among settings of distinct G6PDd variant composition, malaria endemicity, and teams managing the evaluation. Taken together, these real world assessments of the G6PD RDT indicate suitability for intended use in guiding safe access to primaquine therapy against relapse.

In the current study, the G6PD RDT provided a superb margin of safety in the context of G6PD screening for the purpose of reaching a clinical decision on primaquine therapy (i.e., NPV = 100%). The FST resulted in two male subjects being falsely classified as normal despite enzyme activity below 10% of normal (NPV = 99.1%). Deviation from 100% NPV means vulnerable patients will be in danger of exposure to primaquine (Fig 4), and this did occur in the current study with female G6PD RDT testing with NPV = 99.7% (at <30% analysis in one females subject with 22% of normal G6PD), as well as in two of the studies cited above: 97.7% [38], 98.2% [39].

G6PD RDT and FST each showed a propensity for false deficient reads across the spectrum of G6PD activity among subjects, especially females. Test failure to chemically develop may result in falsely deficient outcomes. This apparently occurred at a relatively high rate in this study and others. The DPV of G6PD RDT and FST at a 30% enzyme activity threshold for females, 17% and 18%, reflected this diagnostic problem. In other words, 83% and 82% of female subjects screening as deficient were actually “normal” (>30% G6PD activity). Partial development of color or fluorescence would have prompted test readers to classify lesser color intensity as “deficient”. We viewed this approach as clinically appropriate with respect to preventing exposure to primaquine in patients at risk, i.e. protecting NPV with compromise of DPV. That compromise results in patients who could safely consume primaquine therapy being denied it (Fig 4).

Female heterozygotes present a serious diagnostic problem. In the current study all subjects were evaluated for quantitative G6PD activity, using <5.0 U/gHb to classify each as deficient (with Hb level > 8.0 g/dL). Fig 6 clearly illustrates females almost exclusively occurring in the range of 30% to 60% of normal G6PD activity. They screened as both deficient and normal in that range, largely depending upon placement within that range, precisely as observed in a laboratory-based study of G6PD RDT and FST [30]. Consequently, any normal classification of females by screening may not be considered assurance of safety with primaquine therapy. As expressed by WHO [28], females cleared for primaquine therapy by a normal G6PD screen may nonetheless require clinical monitoring for assurance of safety.

This study employed well-trained laboratorians as readers of the qualitative G6PD diagnostic kits evaluated. This had no impact on the primary objective of this study—examining the performance of the new G6PD RDT relative to the FST standard. Each test likely benefitted equally from the relatively high level of skill of the readers. Thus, while the tests were performed in the setting of a village in the endemic rural tropics, those performing the tests were imported from the setting of a sophisticated modern medical research laboratory. An evaluation of the suitability of the G6PD RDT should be done employing the intended end-users, i.e., paramedics or specially trained residents who today conduct malaria RDT diagnostics and dispense antimalarial therapy at the village level. Proper training on analysis and documentation as well as standard operating procedure must be implemented with use of G6PD RDT by less well trained staff. The use of venipuncture rather than fingerstick blood sample represents another limitation of the study. However, others have demonstrated no difference in G6PD activity estimates from venous versus capillary blood samples [40].

Screening for G6PD deficiency by qualitative point-of-care kits like the first-generation one evaluated here will likely be improved. Nonetheless, in the meantime the present version of G6PD RDT certainly offers an option that is conspicuously better than the current standard of care for most patients with vivax malaria—no G6PD screening and the raw choices of risk of harm by the drug or by the parasite in withholding it. The broad availability of practical and effective kits would vastly mitigate G6PD deficiency as a serious barrier to access to primaquine therapy against relapse.

### Conclusions

This study affirms the good diagnostic performance of a new qualitative G6PD screening device, the G6PD RDT, intended for use at the point-of-care typical of where most malaria patients live. The G6PD RDT always correctly classified male patients with severe G6PD deficiency, whereas the FST failed to do on two occasions—a serious problem imposing risk of harm with primaquine therapy. Both screening kits often misclassified G6PD normal subjects as deficient, which would result in withholding primaquine therapy from patients who could safely consume it. All qualitative tests for G6PD suffer the drawback of classifying many female heterozygotes as G6PD normal despite significantly impaired G6PD activity (i.e., 30% to 60% of normal), exposing them to risk of harm with primaquine therapy. The degree of that risk is poorly understood and requires a great deal more work, both in terms of assessing it and mitigating it with improved diagnostics. There is also a need to evaluate the stability of the RDT during storage in the field.

## Supporting Information

S1 Checklist STARD Checklist. (DOCX)
