# Supplementary material for: Assessment of Point-of-Care Diagnostics for G6PD Deficiency in Malaria Endemic Rural Eastern Indonesia
Source: PLoS Negl Trop Dis. 2016 Feb 19;10(2):e0004457. doi: 10.1371/journal.pntd.0004457 (PMC4760930; doi:10.1371/journal.pntd.0004457)
Supplement: S1 Checklist — (DOCX) [file pntd.0004457.s001.docx]

STARD List

| Section & Topic | No. | Item | Page No and Comment |
| --- | --- | --- | --- |
| Title or Abstract | 1 | Identification as a study of diagnostic accuracy using at least one measure of accuracy (such as sensitivity, specificity, predictive values, AUC) | p.2 “…as the cut-off for qualitative testing, the sensitivity, specificity, positive predictive value, and negative predictive value for G6PD RDT versus FST…” |
| Abstract | 2 | Structured summary of study design, methods, results, and conclusions (for specific guidance see STARD for Abstracts) | p. 2 “This device and the standard qualitative fluorescent spot test (FST) were each compared against the quantitative spectrophotometric assay for G6PD activity…”. |
| Introduction | 3 | Scientific and clinical background, including the intended use and clinical role of the index test | pp.4-5 “…..the most threatening scenario is becoming infected by the parasite P. vivax and being prescribed the drug primaquine to prevent the repeated clinical attacks…”. “….primaquine toxicity in G6PD deficient patients…”. |
|  | 4 | Study objectives and hypotheses | p. 6 “…availability of such robust devices where most malaria patients live is a key to control and elimination of endemic P. vivax malaria”. “The G6PD RDT perfomed as well as the FST”. |
| Methods | 5 | Whether data collection was planned before the index test and reference standard were performed (prospective study) or after (retrospective study). | p. 6 “…work flow where the research team engaged community gathered at churches or other social functions…”. A prospective study. |
|  | 6 | Eligibility criteria | p.6 “After obtaining informed consent of 610 healthy subjects at least 6 years old…”. |
|  | 7 | On what basis potentially eligible participants were identified (such as symptoms, results from previous tests, inclusion in registry). | p.6 “After obtaining informed consent of 610 healthy subjects at least 6 years old…”. |
|  | 8 | Where and when potentially eligible participants were identified (setting, location and dates). | p. 6 “A total of 1117 subjects resided in this village”. “Residents were invited to a study centre established in the village at designated times and dates between April and May 2014”. |
|  | 9 | Whether participants formed a consecutive, random or convenience series. | p.6 |
| Test Methods | 10a | Index test, in sufficient detail to allow replication | p. 7-8 G6PD RDT, Fluorescent Spot Test |
|  | 10b | Reference standard, in sufficient detail to allow replication | p.8 G6PD Quantitative Test |
|  | 11 | Rationale for choosing the referene standard (if alternatives exist). | No alternative except for quantitative test. |
|  | 12a | Definition of and rationale for test positivity cut offs or result categories of the index test, distinguishing pre-specified from exploratory | p.8-9 “..set to G6PD activity <5U/g Hb as deficient”. |
|  | 13a | Whether clinical information and reference standard results were available to the performers/readers of the index test | No, the tests were independently assessed and done by different research assistants. |
|  | 13b | Whether clinical information and index test results were available to the assessors of the reference standard. | No. |
| Analysis | 14 | Methods for estimating or comparing measures of diagnostic accuracy | p.11 “Statistical significance of diagnostic performance indicator by diagnostic test was evaluated by Chi-square test. Sensitivity, specificity, DPV and NPV were presented using proportion analysis and Fisher’s exact 95% confidence intervals. Mean and range of hemoglobin level were calculated to determine distribution by gender. Data were analyzed using Stata 9.” |
|  | 15 | How indeterminate index test or reference standard results were handled. | P. 13 Genotyping |
|  | 16 | How missing data on the index test and reference standard were handled. | p.13 Genotyping, though 1 sample cannot be genotyped. |
|  | 17 | Any analyses of variability in diagnostic accuracy, distinguishing pre-specified from exploratory | p.10-11, 13-14 |
|  | 18 | Intended sample size and how it was determined | Use 95%CI width (0.1), prevalence 10%, expected sensitivity 0.97 |
| Results | 19 | Flow of participants, using a diagram | Fig. 2 |
|  | 20 | Baseline demographic and clinical characteristics of participants | p.6 |
|  | 21a | Distribution of severity of disease in those with the target condition | p. 5 “In South and Southeast Asia, where more than 80% of vivax malaria attacks occur, the extraordinary diversity of G6PDd is dominated by Mediterranean-like, severely deficient variants”. |
|  | 21b | Distribution of alternative diagnoses in those without the target condition | - |
|  | 22 | Time interval and any clinical interventions between index test and reference standard | Not applicable |
|  | 23 | Cross tabulation of the index test results (or their distribution) by the results of the reference standard | Table 3 |
|  | 24 | Estimates of diagnostic accuracy and their precision (such as 95% confidence interval) | Table 3 |
|  | 25 | Any adverse events from performing the index test or the reference standard | Not applicable |
| Discussion | 26 | Study limitations, including sources of potential bias, statistical uncertainty and generalisability | pp. 13-14, 16 |
|  | 27 | Implications for practice, including the intended use and clinical role of the index test | p. 17-18 |
| Other Information | 28 | Registration number and name of registry | No |
|  | 29 | Where the full study protocol can be assessed | No |
|  | 30 | Sources of funding and other support; role of funders | p.19 |
